# Epidemiology, Natural History, Risk Factors, and Prevention of Graves’ Orbitopathy

**DOI:** 10.3389/fendo.2020.615993

**Published:** 2020-11-30

**Authors:** Luigi Bartalena, Eliana Piantanida, Daniela Gallo, Adriana Lai, Maria Laura Tanda

**Affiliations:** Endocrine Unit, Department of Medicine and Surgery, University of Insubria, ASST dei Sette Laghi, Varese, Italy

**Keywords:** Graves’ orbitopathy, Graves’ disease, smoking, radioiodine (131I) treatment, TSH receptor antibodies, hyperthyroidism, hypothyroidism

## Abstract

GO is the most frequent extrathyroidal manifestation of Graves’ disease, although it may rarely occur in euthyroid/hypothyroid patients with chronic autoimmune thyroiditis. It is a relatively infrequent disorder, and men tend to have more severe ocular involvement at an older age. The prevalence of GO is lower than in the past among patients with recent onset Graves’ hyperthyroidism, and moderate-to-severe forms requiring aggressive treatments are no more than 5–6% of all cases of GO. After an initial inflammatory (active) phase and a phase of stabilization (plateau phase), GO tends to improve and eventually inactivates (inactive or burnt-out phase). Minimal-to-mild GO often remits spontaneously, but complete *restitutio ad integrum* almost never occurs when GO is more than mild. Several risk factors contribute to its development on a yet undefined genetic background. Cigarette smoking is the most important of them. Early diagnosis, control and removal of modifiable risk factors, early treatment of mild forms of GO may effectively limit the risk of progression to more severe forms, which have a profound and dramatic impact on the quality of life of affected individuals, and remain a therapeutic challenge, often requiring long-lasting and multiple medical and surgical therapies.

## Introduction

Graves’ orbitopathy (GO), also named Graves’ ophthalmopathy, Thyroid-eye disease, or Thyroid-associated ophthalmopathy, is a relatively rare, but disabling and disfiguring disease of the orbit, in most cases associated with hyperthyroidism due to Graves’ disease (1). GO is a disorder of autoimmune origin, the pathogenic mechanisms of which remain to be fully elucidated (2). The close link of GO with thyroid autoimmune disorders, mainly Graves’ disease, underpin the hypothesis that GO is triggered by immune reactions against one or more antigens shared by thyroid and orbit. The thyrotropin (TSH) receptor is the most likely culprit, supporting an important role of TSH receptor antibodies (TRAbs) in the pathogenesis and course not only of thyroid disease, but also of orbital disease (3, 4). The insulin-like growth factor-1 (IGF-1) receptor seems also to be involved, which may explain the recent promising results obtained by treating patients with active, moderate-to-severe GO with an IGF-1 receptor antagonist monoclonal antibody, teprotumumab (5, 6). This would also be in keeping with a putative, but yet to be proven, protective role of antibodies to the IGF-1 receptor found in serum of patients with GO (7). The histopathologic changes underpinning orbital remodeling (expansion of the fibroadipose tissue, swelling of the extraocular muscles), as well the increased production of hydrophilic glycosaminoglycans by orbital fibroblasts provide a mechanical basis for understanding most of the clinical features of GO (soft tissue changes/inflammation, exophthalmos, diplopia, compressive dysthyroid optic neuropathy) (8).

## Epidemiology

### Incidence

Information on the incidence of GO is scant. In a study of residents in the Olmsted County, Minnesota, through a 15-year interval (1976–1990), 120 incident cases of GO were found, with an age-adjusted incidence of all-degree GO of 16/100,000 population/year for women and 2.9/100,000 population/year for men (9) (**Table 1**). In a more recent prospective multicenter study from Sweden, covering a population of more than 3,500,000 individuals, newly diagnosed cases of Graves’ hyperthyroidism from 2003 to 2005 were 2,200, with an incidence of 21/100,000 population/year; of these, 20.1% had eye involvement of all degrees (4.9% moderate-to-severe), with an overall incidence of GO of 4.2/100,000 population/year (10). Because a 3.9:1 female to male ratio for Graves’ hyperthyroidism was reported in that study, an approximate incidence of 3.3/100,000 population/year in women and 0.9/100,000 population/year in men can be derived for GO of all degrees (**Table 1**). Moderate-to-severe cases of GO would then have an incidence as low as 0.05/100,000 population/year, but classification criteria were not indicated in details (10). In a prospective registry-based study of patients with incident moderate-to-severe GO seen in Denmark during the period 1992–2009, 143 new cases of moderate-to-severe GO, classified following standardized criteria, were registered, with an incidence rate of 1.61/100,000 population/year (2.67/100,000 population/year for women, 0.54/100,000 population/year for men) (11). Figures were similar before and after iodine fortification of salt (11).The Danish study did not consider patients with mild GO (11). However, because about two thirds of Graves’ patients have no or mild GO (13, 14), it was calculated that the incidence of GO of all degrees derived from the Danish study (11) might approximately be 4.83/100,000 population/year (8.01/100,000 population/year in women, 1.62/100,000 population/year in men) (12) (**Table 1**). The considerable differences among the various studies underscore the difficulties in establishing the true incidence of GO. Calculated figures derived from the two more recent studies (10, 11) appear to be lower than those reported in the older and pivotal study from USA (9). These differences might be apparent, due to difficulties in identifying patients with mild GO in registry-based studies (10, 11), or to persistent low referral rate of mild cases of GO, leading to an underestimation of all incident cases. On the other hand, this trend might be real and reflect a true decrease in the incidence of GO in recent years related to several factors, including changes in referral, improved interaction among general practitioners, endocrinologists and ophthalmologists, better control of risk factors, particularly smoking.

**Table 1:** Estimated incidence of Graves’ orbitopathy (GO).

Author (year)	Years of observation	Estimated Incidence in Women	Estimated Incidence in Men	Ref.
Bradley (1994)	1976-1990	16/100,000/year	2.9/100,000/year	(9)
Abraham-Nordling (2011)	2003-2005	3.3/100,000/year	0.9/100,000/year	(10)
Laurberg (2012)	1992-2009	2.67/100,000/year* 8.01/100,000/year**	0.54/100,000/year* 1.62/100.000/year**	(11)

*Moderate-to-severe forms of GO.

**All degrees of GO estimated by assuming that moderate-to-severe forms of GO represent 1/3 of cases (12).

### Prevalence

A recent study from the European Group on Graves’ Orbitopathy (EUGOGO) used reported data on the incidence of GO (10, 11, 15) to estimate the prevalence of GO in the general population in Europe (12). By these calculations, it would appear that the prevalence of GO of all degrees in Europe might be between 90 and 155/100,000 population (12). Although this calculation is based on only three studies, these are large, epidemiological investigations and likely provide sound estimates of the overall prevalence of GO. Previous approximate calculations yielded an estimate of GO prevalence in Europe ranging from 100 to 305/100,000 population (16). The estimated prevalence derived from the Olmsted County, USA study was about 250/100,000 population (9). Other studies [reviewed in Ref. (17)] have led to an estimated prevalence of 100–300/100,000 population in Asia. Thus, although limitations inherent to the way of calculating prevalence from incidence data must be taken into account, it is reasonable to state that prevalence of GO does not substantially differ in different ethnic groups and is comprised between 90 and 300/100,000 population. GO, although relatively infrequent, does not fulfill the main criterion for being defined as a rare disease, i.e., a prevalence <50/100,000 population. However, several variants of the disease, namely, euthyroid GO, GO associated with thyroid dermopathy or acropachy are far below this threshold and can be considered as rare diseases, provided that distinct pathophysiological mechanisms can be identified (12).

**Figure 1:**
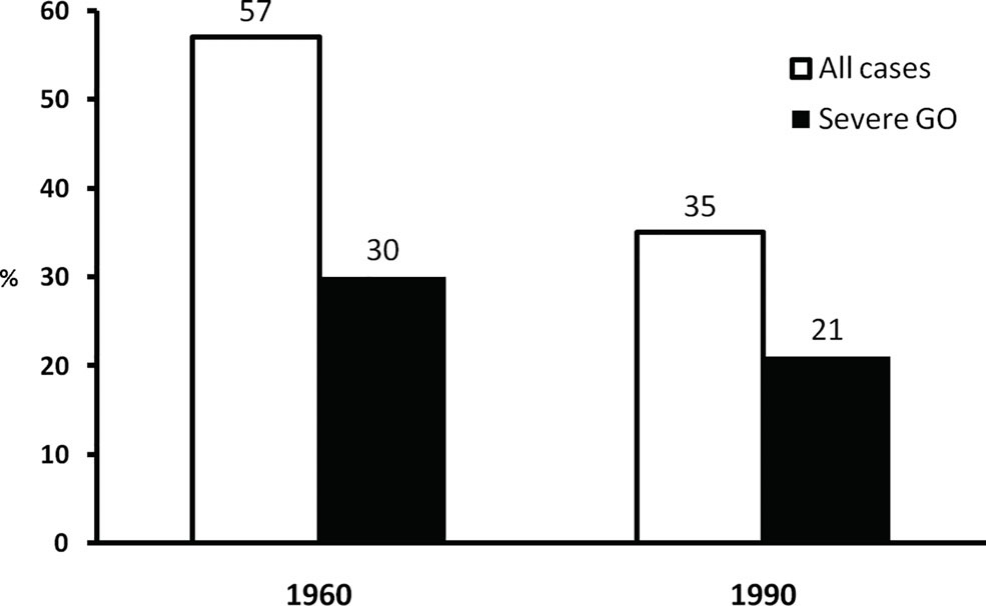
Prevalence and severity of Graves’ orbitopathy (GO) in the first 100 consecutive patients seen in a combined thyroid-eye clinic in UK in 1960 and 1990. Derived from Perros and Kendall-Taylor (18).

**Figure 2:**
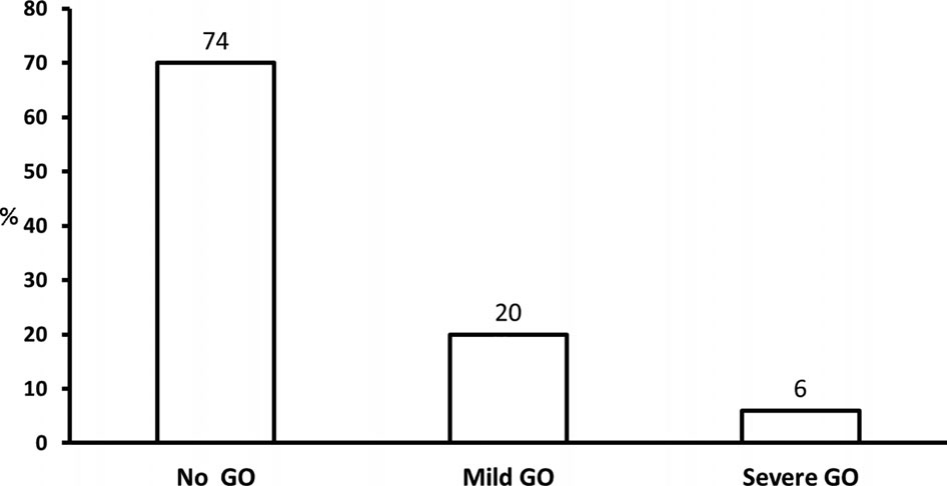
Prevalence and severity of Graves’ orbitopathy (GO) in 346 patients with recent onset and untreated Graves’ hyperthyroidism. Severe GO: moderate-to-severe and sight-threatening GO. Derived from Tanda et al. (13).

Many patients with Graves’ disease have no ocular involvement when first diagnosed with hyperthyroidism. A review of clinical records of the first 100 consecutive patients seen at a tertiary referral center in UK in 1960 and 1990 demonstrated that 57 and 35%, respectively, had clinically relevant GO with a parallel decrease in the proportion of severe forms in the two decades (**Figure 1**) (18) In a large and more recent series of 346 patients with newly diagnosed and recent onset Graves’ hyperthyroidism seen at a single center in Italy, almost three quarters had no ocular involvement, and only 6% had moderate-to-severe GO (including 0.3% with sight-threatening GO) (**Figure 2**) (13). A recent meta-analysis and systematic review of 57 studies including 26,804 patients reported an overall prevalence of 40% (19). It should be noted that included papers likely comprised patients with early onset GO and patients with GO of longer duration. Thus, it can be concluded that the overall prevalence of GO of all grades among patients with Graves’ hyperthyroidism is comprised between 25 and 40%. It is possible that early diagnosis and treatment of hyperthyroidism, as well as use of preventive measures, including removal of modifiable risk factors (see a subsequent section of this manuscript), may reduce the prevalence of clinically significant GO to the lower end of the above spectrum, or even lower. In addition, in those patients who have GO at onset, early referral and management may be associated with a change in the clinical manifestations of GO. In the PREGO (Presentation of Graves’ Orbitopathy) study, patients referred to EUGOGO centers in the year 2012 were compared to patients referred to EUGOGO centers in the year 2000: the 2012 cohort had a shorter referral time and showed a much higher prevalence of mild forms (60.5 *vs.* 41.2%) and inactive forms (63.2 *vs.* 39.9%) of GO when compared with the 2000 cohort (14). Nowadays, moderate-to-severe forms of GO, which remain a major therapeutic challenge, represent 5–6% of cases (11, 13). Although it is difficult to draw definitive conclusions, it seems that the proportion of Graves’ patients with GO of all grades and, particularly, with severe forms of the disease is possibly declining over time (20).

### Age, Gender, and Ethnicity

In the Olmsted County study, GO of all degrees showed a bimodal peak, 40–44 years and 60–64 years in women, 45–49 years and 65–69 years in men (9). In an observational Japanese study of 10,931 consecutive patients, the mean age of GO occurrence was 39 years in women and 43 years in men (21). In a study of 101 consecutive patients referred to a combined thyroid-eye clinic, the mean age was lower in patients without GO (40 years) than in those with GO (46 years) (22). In an Italian study mean age did not differ in Graves’ patients without GO and in those with mild GO (46 and 44 years, respectively), but was significantly higher in patients with moderate-to-severe GO (54 years) (13). Likewise, in a Danish study of patients with moderate-to-severe GO, the median age was 50 and 56 years before and after salt iodization, respectively, and the risk of developing moderate-to-severe GO was lower in patients aged <40 years (11).Thus, age is a relevant factor affecting severity of GO, and the disease tends to be more severe in older patients (22). Although a questionnaire-based survey among European thyroidologists reported the presence of GO of all degrees in approximately one third of juvenile Graves’ disease (23), clinically relevant GO in childhood is in general rarer than in adults and usually mild (24).

GO is more frequent in women than in men, although the female-to-male (F/M) ratio varies in different studies. In a study of 202 consecutive Graves’ patients, the F/M ratio was 3.4 in patients without GO, 2.1 in patients with GO, and 0.7 in euthyroid GO (25). Other studies reported F/M ratios of 3.9 (21) and 4.2 (10). Gender affects also severity of GO, the F/M ratio progressively decreasing with increasing severity of GO (22). Likewise, in a cohort study of 2045 Graves’ patients, although the proportion of patients with clinically relevant GO (NOSPECS class ≥2) was similar in women and men (51.5 and 52.7%, respectively), patients with more severe GO (NOSPECS class 4–6) were more frequently men (30.4 *vs.* 21.3%, p <0.001), and their median age was also higher than in women with similar severity of GO (52 years *vs.* 40 years, p <0.05) (27). Although a registry-based Danish study failed to show any significantly different risk of developing moderate-to-severe GO in men and women (11), it seems reasonable to conclude that GO tends to be relatively more frequent and severe in men, in whom it occurs at a more advanced age.

Relevance of ethnic factors in the occurrence of GO is controversial. In a study of 155 patients with newly diagnosed Graves’ disease (116 Caucasians and 39 of Asian origin), the prevalence of GO was significantly higher in Caucasians than in Asians (42 *vs.* 7.7%, p = 0.0002), with a risk of developing GO 6.4-fold higher in Caucasians, possibly in relation to the high prevalence of smokers (>60%) among Caucasians (28). Conversely, a recent meta-analysis and systematic review showed a slightly lower prevalence of GO among Caucasians (37%) compared with Asians (45%) (19). A study from Australia reported that Caucasians have an increased risk (2.08, 95% CI, 1.56–2.76) of developing GO compared with non-Caucasian ethnicities (29). Conversely, other studies failed to link ethnic origin (including Caucasians, African Americans, Asian Americans, Asians, Latinos) to an increased for the occurrence of GO (30, 31). In summary, the role of ethnic factors, if any, remains, for the time being, unclear.

### Phenotype

**Figure 3:**
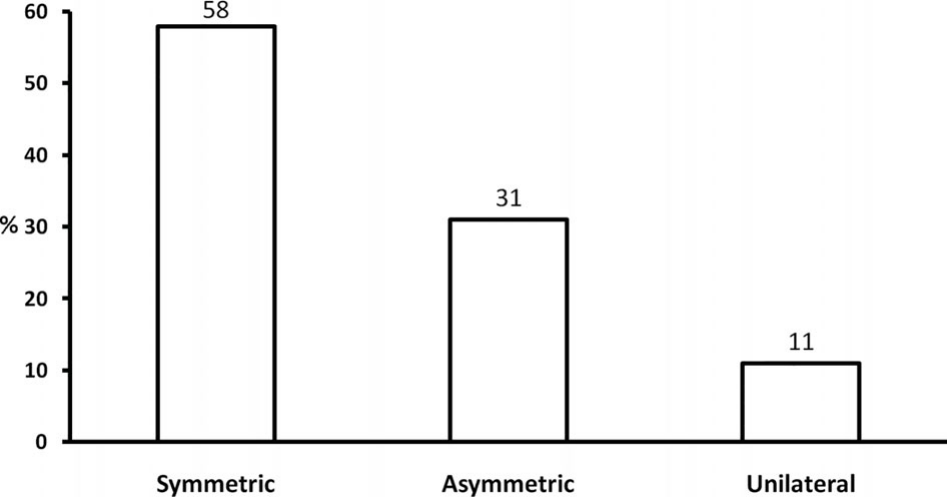
Presentation of Graves’ orbitopathy in a series of 269 patients referred to EUGOGO tertiary centers. Derived from Perros et al. (26).

The majority of patients affected with GO have bilateral disease, but asymmetric or even unilateral GO may also develop (1). Asymmetric forms have been described in 4–14% of cases, unilateral forms in 9–34% of cases (9, 32–34). In a recent multicenter study of 269 patients referred to EUGOGO centers, although the majority (157 patients, 58%) had symmetric GO, many patients had either asymmetric or unilateral GO (26) (**Figure 3**). Interestingly, patients in the asymmetric group were older, were more frequently men, and tended to have more severe and active GO than the other groups (26). The reasons for asymmetric presentation of GO are unknown, but they might include differences in the anatomy of bony orbit or its vascularization. One study showed that patients with euthyroid/hypothyroid GO tend to have milder and more asymmetric forms of the disease (35). On the other hand, unilateral or asymmetric GO may sometimes progress to bilateral disease (34, 36). Asymmetric and unilateral forms of GO require an accurate diagnostic assessment to exclude other orbital diseases mimicking GO, reviewed in Ref. (37). Graves’ disease may be an isolated disease or associated with other autoimmune disorders in the context of Autoimmune Polyglandular Syndromes: prevalence and severity of GO in these two different settings do not differ (38).

### Relation With Thyroid Dysfunction

Most patients affected with GO are hyperthyroid (1), but a substantial proportion, ranging from 0.2 to 11% in different studies (35, 39–41), are euthyroid or subclinical/overt hypothyroid. TSH-receptor antibodies (TRAbs) are the ultimate responsible for hyperthyroidism due to Graves’ disease (42), but they also strongly correlate with the clinical activity and severity of GO (3, 4, 43). But, in a series of 700 unselected patients with chronic autoimmune thyroiditis, overt GO was present in 44 (6%): among them TRAb bioassay for stimulating activities tested positive in 30/44 (68%) patients with GO *vs.* 36/656 (5.5%, p <0.001) patients without GO (44). Thus, it is conceivable that TRAbs can explain the rare occurrence of GO in patients without hyperthyroidism. A note of caution should be added by mentioning that cases of so-called euthyroid/hypothyroid GO might indeed be a manifestation of another emerging disease, IgG4 ophthalmic disease, which has clinical features similar to GO (45). This underscores again the need for a careful differential diagnosis in doubtful cases.

A close relationship exists between the onset of Graves’ hyperthyroidism and the onset of GO, because in about 85% of cases GO develops within 18 months before or after the onset of hyperthyroidism (25, 46). Therefore, a normal thyroid status does not exclude the diagnosis of GO, because hyperthyroidism may occur long after the development of GO. On the other hand, when GO follows hyperthyroidism, a relevant question is whether treatment for hyperthyroidism can affect the development and course of GO (47). Hyperthyroidism *per se* negatively influences the course of GO, and restoration of euthyroidism is often associated with a stabilization/improvement of GO (48). But hypothyroidism can have a negative impact on the occurrence/progression of GO as well (45, 46). Thus, restoration and maintenance of euthyroidism is fundamental. While antithyroid drug treatment and thyroidectomy are apparently neutral to the course of GO, radioactive iodine (RAI) treatment is associated with a small, but definite risk of progression or *de novo* occurrence of GO, especially in smokers (49) (see a subsequent section).

### Natural History

Following the initial description by Rundle and Wilson (50), it is widely accepted that overt GO clinically goes through an initial phase of inflammatory changes corresponding to ongoing activation of the pathogenic cascade of events occurring in the orbit (active phase); the disease then stabilizes when inflammation starts to subside (plateau or static phase), and then progressively improves in association with burning out of inflammation (inactive phase), without going back to normal (51). It is unknown how long it takes for GO to become inactive, but it is generally believed this occurs within 18–24 months. It should be mentioned that, although rarely, late reactivation of GO may occur (52). In a computer tomography-based study, the increase in the extraocular muscle volume appeared to be an early phenomenon, while the increase in the orbital fat volume occurred later (53). A precise definition of the natural course of GO of all degrees is, however, hampered by the fact that patients with moderate-to-severe and active GO cannot be left untreated and are, therefore, given disease-modifying treatments, namely intravenous glucocorticoids, orbital radiotherapy (54) or, more recently, biological agents (2).

**Figure 4:**
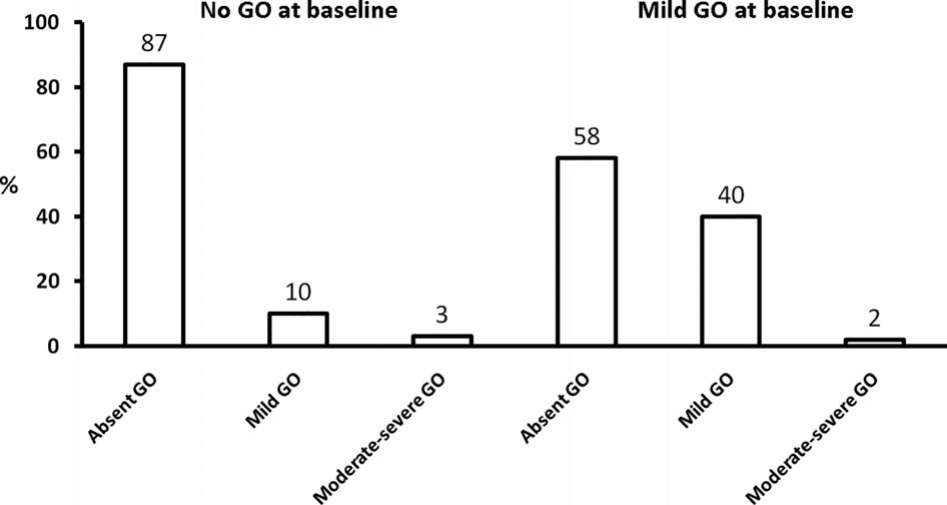
Natural history of Graves’ orbitopathy (GO) at the end of antithyroid drug treatment according the absence (n = 194) or presence (n = 43) of mild GO at baseline. Derived from Tanda et al. (13).

In a series of 59 patients with untreated GO referred to a combined thyroid-eye clinic in UK, 13 patients (22%) improved substantially, 25 patients (42%) manifested only slight amelioration, 13 patients (22%) were stable, and 8 patients (14%) had a progression of GO over a median period of 12 months (55). In series of 196 Graves’ patients, 81 (41%) developed GO, 53 of whom (65%) received no treatment, except for local measures: 25/53 (47%) improved substantially, 26/53 (49%) had unchanged GO at follow-up, 2/53 had a progression of GO (56). In a large series of 346 recent onset and untreated Graves’ patients, 237 then completed a course of antithyroid drugs; 194 had no GO at baseline, and progression to moderate-to-severe GO occurred in <3% of cases; among the 43 patients with mild and inactive GO at baseline, progression to moderate-to-severe GO occurred in only 1 patient, while the majority of patients had a complete remission of ocular manifestations (13) (**Figure 4**). In another series of 65 patients, the large majority of whom had mild, minimally active GO and were followed without treatment for a median of 40 months, 51% improved spontaneously, 34% remained stable, 15% had some progression of GO (57). In summary, it would appear that mild/minimal GO rarely progresses to severe forms, particularly if hyperthyroidism is adequately managed and controlled, while stabilization and remission is frequent in mild forms of GO (20). This favorable course of mild GO is favored by control of modifiable risk factors (see a subsequent section). No conclusion can be drawn on the natural history of moderate-to-severe GO, because the latter is promptly treated by disease-modifying therapies. However, in a retrospective study of 226 patients with initially moderate-to-severe and active GO reexamined after a median of about 4 years after GO onset and various non-surgical and surgical treatments for GO, further amelioration was observed in 60% of responders to treatments at the last visit (58). This suggests that time (i.e., the natural history) is a key factor affecting also the long-term outcome of GO after treatment (58). In addition, in the placebo group of the second teprotumumab study, at the end of 6 months of observation a reduction in exophthalmos ≥2 mm was observed in 10% patients, an amelioration in diplopia in 29%, and a CAS 0-1 (inactive GO) in 21% (6). This suggests that the Rundle’s curve description applies also to patients with moderate-to-severe GO. Obviously, this by no means implies that in this category of patients therapies should be postponed, because early immunosuppressive treatments improve and inactivate GO, thereby affecting substantially the natural history of GO, and reduce the time interval required to proceed to rehabilitative surgery, if needed.

## Risk Factors and Prevention

GO results from a complex interaction of endogenous (unmodifiable) and exogenous/environmental (modifiable) risk factors. The former include age, gender, and genetic factors. As described in a previous section of this manuscript, GO tends to be more severe in men, in whom it occurs at an older age than in women. A search for genetic factors did not provide unequivocal information, with particular regard to differentiation between Graves’ patients with or without GO (59–61). Likewise, results are conflicting as to the association between GO and Major Histocompatibility Complex (MHC), cytotoxic T-lymphocyte-associated antigen-4 (CTLA-4), protein tyrosine phosphatase or non-receptor type 22 (PTPN22), interleukins, intercellular adhesion molecule 1 gene polymorphisms, adipogenesis-related genes, TSH receptor or thyroglobulin (17, 62, 63).

Several modifiable risk factors for the occurrence/progression of GO have been identified. This is a very important issue, because controlling them may positively affect the course of GO (64) (**Table 2**).

**Table 2:** Risk factors for the occurrence/progression Graves’ orbitopathy (GO) and preventive actions.

Risk Factor	Evidence (Ref.)	Preventive Action
Smoking Habit	• Patients with GO are more frequently smokers than those without GO (67) • Smokers have an increased risk of developing GO than non-smokers (68) • Smokers have a dose-dependent increased relative risk of developing severe GO (69) • Smokers have a lower and slower response to immunesuppressive treatments (70, 71), • Refrain from smoking reduces the risk of developing exophthalmos and diplopia (69) • Radioiodine associated progression og GO is more frequent in smokers (70, 72),	Urge patients to refrain from smoking
Thyroid Dysfunction	• Hyperthyroidism negatively influences GO and amelioration is associated with restoration of euthyroidism (48) • GO may develop during uncontrolled hypothyroidism (73)	Restore and stably maintain euthyroidism by antithyroid drugs (hyperthyroidism) or levothyroxine replacement (hypothyroidism)
Radioiodine treatment for hyperthyroidism	• Radioiodine treatment may cause occurrence or progression of GO (49, 74), particularly in smokers (72)	Give a short course of oral prednisone (steroid prophylaxis) in at risk patients
Oxidative stress	• GO is associated with an increased oxidative stress (75)	Provide a 6-month selenium supplementation in patients with mild GO of short duration
TSH receptor antibodies (TRAbs)	• TRAb levels are higher in patients with GO than in those without GO (76) • TRAb levels correlate with GO activity and severity (4, 43, 76),	In patients with relevant GO, control hyperthyroidism with antithyroid drugs, because this is usually associated with a decline in TRAb levels
Hypercholesterolemia	• Serum total and LDL cholesterol levels correlate the presence and activity of GO (77) • Serum total and LDL cholesterol levels are higher in patients with GO than in those without GO (78) • The use of statins was associated with a decreased risk of developing GO (31)	Correct dyslipidemia in patients with newly diagnosed Graves’ hyperthyroidism

### Smoking

**Figure 5:**
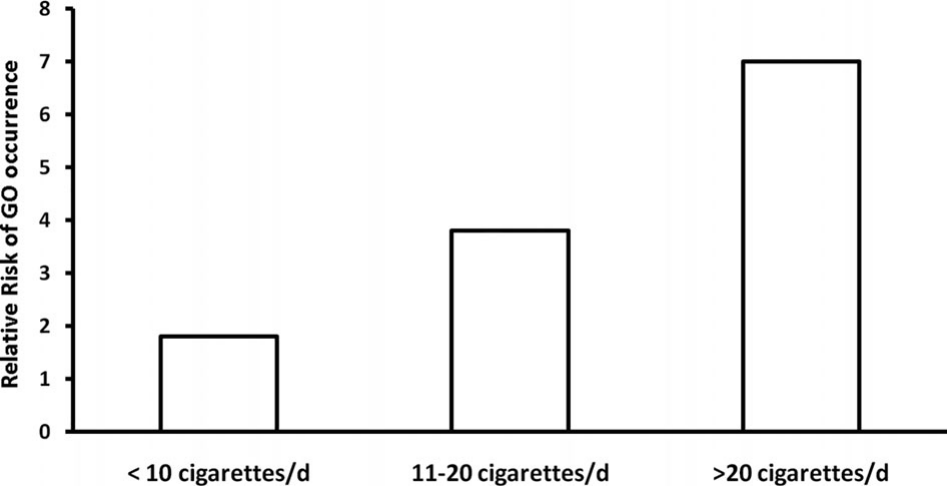
Relative risk of developing exophthalmos and diplopia in relation to daily number of cigarettes. Derived from Pfeilschifter and Ziegler (67).

Smoking habit is probably the most important modifiable risk factor for GO (65, 66) (**Table 2**). The negative impact of smoking on GO is based on the following evidence: i) Graves’ patients with GO are more frequently smokers (and heavy smokers) than those without GO, or patients with other thyroid disorders, including chronic autoimmune thyroiditis (67); ii) Among Graves’ patients, smokers have a higher risk of developing GO than non-smokers (odds ratio, 7.7, 95% CI, 4.3–13.7, *vs.* 1.9, 95% CI, 1.1–3.2) (68); iii) Smokers are at high risk of developing severe forms of GO, with a dose-dependent relative risk of diplopia or exophthalmos (69) (**Figure 5**); iv) Response to treatments for GO is decreased and occurs later in smokers than in non-smokers (70, 71); v) *De novo* development or progression of GO after RAI treatment occurs more frequently in smokers (29, 49, 72); vi) Quit smoking decreases the risk of developing exophthalmos and diplopia, suggesting that current smoking is more important than lifetime tobacco consumption (69). Accordingly, refrain from smoking is a fundamental, general, preventive action strongly recommended by guidelines (54). It is possible, but yet unproven, that also passive smoking be relevant for the occurrence of GO (23). Whether e-cigarette may also concur to development/progression of GO remains for the time being unsettled. Mechanisms whereby cigarette smoking exerts a negative effect on GO are not fully understood, but they might involve oxygen free radical generation, hypoxia in the orbit, enhanced production of cytokines, stimulation of adipogenesis (64).

### Thyroid Dysfunction

We mentioned above that both hyperthyroidism and hypothyroidism represent risk factors for the occurrence of GO (46, 48). Accordingly, a rapid restoration of euthyroidism and its stable maintenance, avoiding thyroid status fluctuations, are strongly recommended by guidelines as an important preventive measure (54) (**Table 2**). Mechanisms whereby thyroid dysfunction may favor the occurrence or progression of GO are probably related to the activation of the TSH receptor by either TRAbs (hyperthyroidism) or TSH (hypothyroidism) (64).

### Radioiodine Treatment

RAI is a well-established and effective method of treatment for Graves’ hyperthyroidism (79). RAI carries a small but definite risk of causing progression or *de novo* occurrence of GO, probably due to antigen release and related exacerbation of autoimmune reactions following RAI administration (1) (**Table 2**). In a randomized clinical trial of 168 patients, 114, aged 35 to 55 years, were randomized to either antithyroid drug treatment, RAI treatment, or subtotal thyroidectomy: GO developed or worsened in 4/38 patients (10%) treated with antithyroid drugs, 6/37 patients (16%) treated surgically, and 13/39 patients (33%) treated with RAI (74). In a randomized clinical trial of 443 patients, GO progressed in 23/150 patients (15%) treated with RAI, but in only 3/148 patients (3%) treated with antithyroid drugs (49). RAI-associated progression of GO was transient in some patients, but was permanent and required immunosuppressive treatments in 5% of patients (49). This untoward effect could be prevented in a third group of patients who received a concomitant short-term course of oral prednisone (steroid prophylaxis) (49), confirming a previous randomized clinical trial from the same group (80). This undue effect of RAI is more likely to occur in smokers (72), while it is unlikely in patients whose Graves’ disease duration is >5 years (81) or whose GO is stably inactive (73). Relatively lower doses of oral prednisone (0.1–0.2 mg/bodyweight as starting dose, gradually tapered and withdrawn after 6 weeks) are equally effective as previously suggested highly doses (0.3–0.5 mg/bodyweight as starting dose, tapered and withdrawn after 3 months) (82) and safe (83). Based on the above studies, steroid prophylaxis is an important preventive measure in most Graves’ patients undergoing RAI treatment for hyperthyroidism, and as such it is recommended in most patients by recent guidelines (54, 79). In particular, it should be given in patients who have mild and active GO and/or risk factors for its development/progression, especially smoking, while It can be avoided in patients with long-standing and inactive GO.

### Oxidative Stress

Graves’ orbitopathy is associated with an increased oxidative stress (75). Because of its antioxidant and immunoregulatory actions, selenium has been proposed as an adjuvant therapy in patients with mild GO. In a randomized clinical trial of 159 patients with mild and active GO, selenium administration for 6 months was associated with an improved quality of life and improved ocular involvement compared with placebo; in addition, it was more effective than placebo in preventing progression of GO from mild to moderate-to-severe (84). It is unclear whether selenium should be used only in patients who are selenium-deficient or also in patients with an adequate selenium intake. It is probably useless in patients with long-standing, inactive mild GO. Likewise, there is no evidence that a course of selenium may be beneficial also in patients with moderate-to-severe GO. With these limitations, current guidelines recommend a 6-month selenium supplementation in patients with recent onset, mild GO, because it improves ocular involvement and prevents progression to more severe forms of GO (54).

### TSH-Receptor Antibody Levels

TRAbs are the only specific biomarker for Graves’ disease and GO (3). TRAb levels correlate with the Clinical Activity Score (4, 43). TRAbs with stimulating activities in a recently developed bioassay tested positive in 150/155 patients with Graves’ disease (97%) and 148/155 of those with GO (95%), their levels were 3-fold higher in patients with GO than in those without GO, and strongly correlated with GO activity and severity (76). TRAbs were shown to be an independent risk factor for GO and predictors of severity and outcome of the disease (85). In a study of 100 consecutive patients, the combination of high TRAb levels and absent thyroid peroxidase antibodies identified Graves’ patients at high risk of developing GO (86). This finding was subsequently confirmed by a 3-year prospective study from the same group (87). In terms of prevention, for the time being, there is no treatment that blocks TRAb synthesis. However, while RAI treatment for hyperthyroidism is followed by a rise in TRAb levels (probably related to the cytolytic effect of RAI), which may last for several years, antithyroid drug treatment (either directly or through restoration of euthyroidism) and thyroidectomy are generally and gradually associated with a progressive reduction of serum TRAb concentration, which may be beneficial for GO (88).

### Hypercholesterolemia

In a cross-sectional study of 250 patients with Graves’ hyperthyroidism of recent onset, with (n = 133) or without GO (n = 117), a correlation between serum total and LDL cholesterol and the presence and activity of GO was reported (77). A second study, from the same group, of 86 consecutive patients with recent onset Graves’ disease referred for RAI treatment, confirmed that serum total and LDL cholesterol were higher in patients than in those without GO, although there was no relationship between cholesterol and activity or severity of GO (78). The above reports might explain why the analysis of a large database from USA, including 8,404 patients with newly diagnosed Graves’ disease, showed that the use of statins for at least two months in the year of observation was associated with a 40% decreased hazard ratio (HR) of developing GO (HR: 0.60, CI, 0.47–0.75) (31). Because the use of non-statin cholesterol lowering drugs was not associated with a decreased risk of GO occurrence (31), it is unsettled whether the purported protective/preventive effect of statins on GO is related to their anti-inflammatory action or to the cholesterol lowering effect. Clearly, further studies are needed to clarify this issue, but it is reasonable to state that correction of dyslipidemia in newly diagnosed Graves’ patients may represent a useful preventive action against the occurrence of GO.

### Prediction of GO Occurrence in Newly Diagnosed Graves’ Patients

Because of the major role of exogenous factors in the development of GO, a prospective observational study was carried out in 10 EUGOGO centers to construct a predictive score (called PREDIGO, prediction of GO) for the development/progression of GO in 348 newly diagnosed and untreated Graves’ patients without obvious GO (89). Four independent variables composed the PREDIGO score, i.e., the Clinical Activity Score, TRAbs (measured as TSH-binding inhibiting immunoglobulins, TBII), duration of hyperthyroidism, and smoking. Of the 348 patients, all submitted to antithyroid drug treatment, 53 (15%) developed GO, which was mild in 46 (13%) and moderate-to-severe in 7 (2%) (78). The PREDIGO score proved to be much better in identifying patients who will not develop GO (negative predictive value: 0.91, 95% CI, 0.87–0.94) than those who will (positive predictive value: 0.28, 95% CI, 0.20–0.37) (89). Although this represents an evident limitation and underpins the need for the improvement and refinement of this score with additional biomarkers, the PREDIGO score may represent a simple and useful tool to consider when discussing the general treatment plan of newly diagnosed Graves’ patients with absent or very mild GO. Moreover, as mentioned earlier, early referral of patients with mild and active GO and/or effective control of modifiable risk factors for development/progression of the disease may substantially contribute to exacerbation of GO (54).

## Concluding Remarks

GO is the most frequent extrathyroidal manifestation of Graves’ disease, although it may rarely develop also in euthyroid/hypothyroid patients with chronic autoimmune thyroiditis. It is a relatively infrequent disorder, and men tend to have more severe ocular involvement at an older age. After an initial inflammatory (active) phase and a phase of stabilization (plateau phase), GO tends to improve and eventually inactivates (inactive or burnt-out phase). Mild GO tends spontaneously to remit spontaneously, but complete *restitutio ad integrum* almost never occurs when GO is more than minimal-to-mild. Several risk factors contribute to its development on a yet undefined genetic background. Cigarette smoking is the most important risk factor. Early diagnosis, control and removal of modifiable risk factors, early treatment of mild forms, stable control of thyroid dysfunction may effectively limit the risk of progression to more severe forms of GO, which have a profound and dramatic impact on the quality of life of affected individuals, and remain a therapeutic challenge, often requiring long-lasting and multiple medical and surgical therapies.

## Author Contributions

All the authors participated in searching literature, critically read the papers, critically revised the draft of the paper, checked illustrations, checked bibliography, and share responsibility for statements. All authors contributed to the article and approved the submitted version.

## Funding

This study was partly supported by funds from the University of Insubria (Fondi d’Ateneo per la Ricerca, FAR) to LB, EP, and MT.

## Conflict of Interest

The authors declare that the research was conducted in the absence of any commercial or financial relationships that could be construed as a potential conflict of interest.
